# Structural T‐Cell Receptor Analysis in the Age of Machine Learning

**DOI:** 10.1111/imr.70168

**Published:** 2026-09-05

**Authors:** Nele P. Quast, Matthew I. J. Raybould, Charlotte M. Deane

**Affiliations:** ^1^ Oxford Protein Informatics Group, Department of Statistics University of Oxford Oxford UK

## Abstract

The development of highly accurate deep learning models for protein structure prediction has transformed the landscape of T‐cell receptor (TCR) structure data, which can now be accessed at repertoire scale. We provide a perspective on the growing field of structural TCR immunoinformatics, summarizing core principles of TCR structural biology and highlighting existing resources and tools. We outline computational methods for TCR structure prediction, and discuss outstanding challenges faced by current tools, as well as potential avenues to address these. We expand on the research enabled by the availability of predicted TCR structures, exploring the utility of TCR structure predictions for computationally inferring TCR specificity, as well as summarizing opportunities emerging from the adjacent field of antibody research. Finally, we provide a forward‐looking perspective on the advances in deep learning research which have recently enabled computational design of TCRs and TCR‐like binders.

## 1 Introduction

The T‐cell immune response is a central component of adaptive immunity, governed primarily by the recognition of peptide–major histocompatibility complex (pMHC) antigens by T‐cell receptors (TCRs) [1]. TCRs are of considerable interest across several areas of biomedical research: they can act as anti‐cancer [2, 3] and anti‐pathogen agents [4], play a role in mediating the immunogenicity of biologic therapeutics [5, 6], and are implicated in autoimmune pathology [7]. Efficiently decoding which pMHC a TCR recognizes is critical both to unlocking their therapeutic potential and understanding their role in disease.

The vast majority of available TCR specificity information comes from library‐based assays, whereby screening can assess the specificity of a single TCR against millions of pMHC dextramer antigens, millions of TCRs against a single pMHC target, or many TCRs against multiple pre‐determined antigen targets using multiplexed approaches [8, 9, 10]. Despite the high‐throughput of TCR‐specificity assays, the potential diversity of TCRs and antigens, estimated to be 10^8^ and 10^15^ for any individual respectively, dwarfs the available specificity data [11, 12, 13, 14]. Therefore, substantial research effort has been dedicated toward distilling the rules governing TCR antigen recognition into computational algorithms that can predict a TCR's complementarity [15, 16].

The dominant approach to predict TCR:pMHC complementarity is to leverage the sequence data from screening campaigns, curated in databases such as VDJdb [14]. However, learning on sequence properties alone has proven to be prone to overfitting, and these methods fail to generalize to unseen antigens, a task which is also referred to as “de‐novo” specificity prediction [17, 18]. Recent estimates suggest that at least a 1000‐fold increase in sequence‐based specificity data would be required to attain computational models capable of generalizing beyond the experimental data [19].

Given the outstanding challenges faced by sequence‐based TCR‐specificity prediction, structure‐based approaches have been gaining traction [20, 21, 22]. These methods acknowledge that antigen recognition is governed by the three‐dimensional (3D) structure and physicochemical complementarity of the TCR:pMHC interface and seek to learn more generalizable properties from the structure of the interface [15, 23]. However, obtaining structure data is expensive and laborious, and existing structure data is relatively sparse [24, 25].

Encouragingly, recent advances in protein structure prediction have begun to alter the landscape of 3D data availability. Deep learning methods, most notably AlphaFold2 and its successors, can now predict protein structures with near‐experimental accuracy for many proteins [26, 27, 28, 29, 30, 31, 32, 33], and have spurred the development of TCR‐specific algorithms capable of modeling immune receptor structures at high throughput [21, 34, 35, 36, 37]. Computational structure predictors can be used to transform sequence datasets into structure datasets, with the potential to uncover orthogonal information conducive to improved understanding of TCR engagement of antigen [25, 38]. Preliminary indications from the IMMREP25 TCR specificity prediction challenge suggest that structure‐informed approaches can exhibit a statistically significant ability to “generalize” to unseen epitopes, bolstering hope that learning on 3D representations might mitigate overfitting [39].

In light of the field's growing maturity, here we provide a perspective on the current state of structural TCR immunoinformatics. We begin by reviewing TCR structure resources, including databases and software, as well as the core principles of TCR structural biology that we and others have learned by studying them. We then discuss computational methods for predicting TCR and TCR:pMHC complex structures, examining what they can reveal about TCR function, the latest research in structure‐based specificity prediction, and outstanding challenges in structure prediction. Finally, we outline forward‐looking perspectives on the science unlocked by the availability of TCR structure data, including at repertoire scales, drawing on adjacent advances in the antibody field, and present the cutting‐edge advances in deep learning research which are beginning to enable in‐silico design of TCRs and other pMHC binding modalities.

### 1.1 T‐Cell Receptors and the Foundations of Their Diversity

T cells are lymphocytes which mature in the thymus and circulate in the blood and secondary lymphatic tissues. They are activated when their membrane‐bound TCRs recognize a cognate antigen on an antigen‐presenting cell. This recognition is mediated by non‐covalent interactions between the TCR and the pMHC, which transduces intracellular signaling in the T cell, activating multiple response pathways of the adaptive immune system. TCRs are the drivers of antigen recognition by T cells, and each T cell presents hundreds, if not thousands, of copies of a single TCR on its surface.

The majority of TCRs consist of an alpha (α) and a beta (β) chain, the sequences of which are predominantly determined by somatic recombination of the variable (V) and joining (J) gene segments from the TCRα (TRA) and TCRβ (TRB) loci respectively [1]. In the case of the β chain, an additional diversity (D) gene segment is inserted in between the V and J sections. Independent pairing of the α and β chains further diversifies the repertoire of TCRs, estimated at > 10^15^, although individuals express a smaller repertoire of 10^6^–10^8^ unique TCRs [12]. In any individual, the distribution of TCR sequences is long‐tailed, that is, it is likely that any particular TCR sequence within a repertoire is unique to an individual T cell, although a subset of TCRs can be vastly enriched to up to thousands of T cells. These expanded clones are indicative of prior T cell activation, differentiation and expansion, in response to immune stimuli [40].

Gamma‐delta (γδ) TCRs are presented by a smaller set of T cells, and are typically enriched in peripheral tissues [41]. Though their role in the immune system has not yet been fully elucidated, they have been linked to surveillance functions and tissue repair [41, 42]. In this review we focus solely on αβ TCRs (henceforth TCRs), which engage pMHC antigen, rather than γδ TCRs, which respond to distinct antigens [42].

**FIGURE 1:**
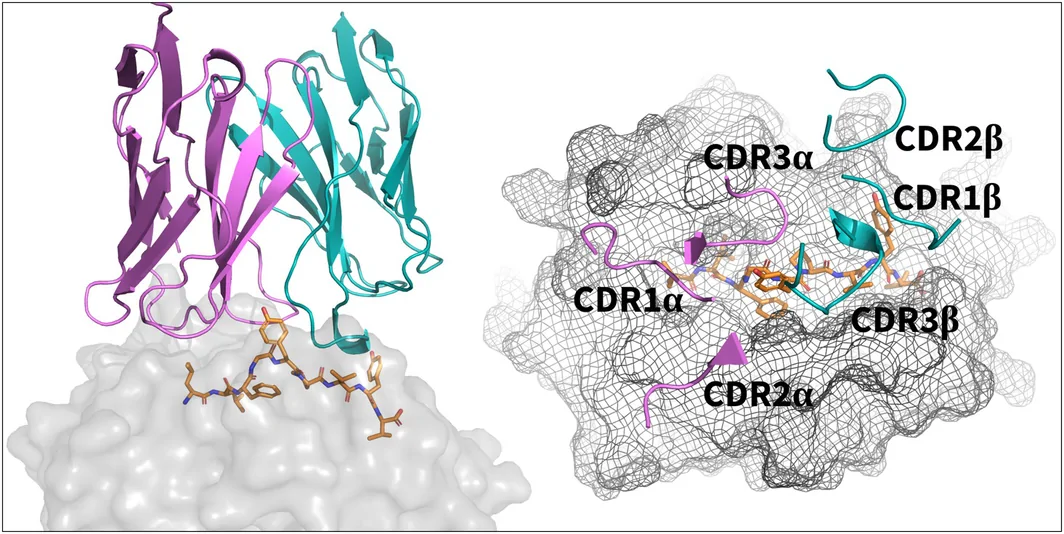
Lateral and top‐down view of a TCR:pMHC structure (PDB code 1AO7 [21]). TCR CDR loops contact the peptide and the MHC in a diagonal orientation, with the TCR*α* chain over the peptide amino‐terminal and the TCR*β* chain over the peptide carboxy‐terminal. TCR*α* magenta, TCR*β* chain teal, peptide orange, MHC Class I gray.

Structurally, TCRs resemble the Fab region of antibodies, although antibodies recognize a broader class of antigen. Each chain of the TCR folds into two compact immunoglobulin domains: a variable and a constant domain. The variable region is often abbreviated to Fv and contains the aforementioned V(D)J‐recombined sequence. The paratope of the TCR is formed by six complementarity‐determining regions (CDRs), three on each chain, that sit at the tip of the Fv with maximal accessibility to pMHCs presented on other cells (Figure 1).

CDR1 and CDR2 are germline‐encoded by the V genes, while V(D)J recombination, in addition to N‐ and P‐nucleotide mutations at the joining sites of the fragments, leads to high sequence diversity in the CDR3α and CDR3β [1, 43]. TCR CDRs are usually defined using structurally aligned numbering schemes, such as IMGT [44]. These define a consistent numbering for TCR chains of variable length. TCR CDR3 loops typically range from 8 to 13 amino acids, while CDR1 and CDR2 loops are typically 5–8 amino acids long [45]. The CDR loop positions are stabilized by a disulfide bond across beta strands between conserved cysteines at IMGT positions 23 and 104.

Peptides presented by the MHC form a major subset of TCR's antigens, which in humans are also referred to as human leukocyte antigen (HLA). These peptides are digested during the normal degradation process of proteins by the proteasome, as well as by the specialized immunoproteasome. This enables TCRs to stimulate an immune response against antigen derived from intracellular processes. MHC molecules are categorized as Class I or Class II and are transcribed by the highly polymorphic gene complex known as the MHC. Both MHC Class I and MHC Class II molecules are heterodimers which form a peptide‐binding cleft between two alpha helices [46, 47].

Peptides bind to this MHC cleft via “anchor” residues, which largely determine the compatibility of the peptide with a given MHC molecule. In class‐I MHC these are typically residue positions two and nine. Given the length of class‐II‐presented peptides and the open binding groove, these peptides can have different “registers,” that is, shifted positions, at which they are presented, provided the residue side‐chains are sufficiently complementary to the MHC binding pockets along the length of the groove. The residues that sit in the MHC binding pockets largely determine whether a peptide can be presented, while, in general, the identity of the other amino acids can vary without effecting antigen presentation [48, 49].

To ensure a minimum binding affinity to foreign antigens while preventing autoimmune responses, T cells undergo positive and negative selection in the thymus [50]. Immature CD4+ and CD8+ “double‐positive” T cells, which originate in the bone marrow and express both co‐receptors, have a short half‐life unless they are rescued by recognizing self‐peptide MHC in the thymus [51]. Positively selected T cells mature into single‐positive CD4+ or CD8+ T cells. To prevent autoimmune reactions, T cells also undergo negative selection in the thymus, whereby any TCRs with high affinity for self‐antigen trigger apoptosis of the T cell [52]. It is an active area of research to establish fine‐grained details of how positive and negative selection are differentiated within the thymus, given that both processes rely on TCR to self‐pMHC binding. The leading broad‐scale hypothesis is that the selection mechanism is based on the half‐life of the bound TCR complex, which in turn depends on the TCR's affinity to the antigen: low levels of affinity are required for positive selection, while high levels of affinity lead to negative selection [53, 54].

As such, TCR recognition of pMHC is finely tuned, with binding affinities in the micro‐molar range, substantially weaker than typical antibody:antigen affinities [55]. The phenomenon of somatic hypermutation in B‐cell receptor repertoires, whereby further sequence diversification beyond V(D)J recombination increases the affinity of mature antibodies, does not act on natural T‐cell receptor repertoires. Reflective of this, slight changes in the amino acid sequence of the binding site are sufficient to change the binding and signaling outcome of a TCR [56].

After discovery of a natural TCR complementary to an antigen of interest, researchers frequently explore sequence mutations to increase affinity to the target; this is key for many diagnostic or therapeutic applications, such as when converting TCRs into highly specific pMHC binding arms within soluble biologic formats. Such formats include non‐TCR pMHC binders, so called TCR‐mimetics (TCRms), often bispecific antibodies [57], though other formats, such as helical mini‐binders, have also been explored [58, 59, 60]. Additionally, several labs and companies have created artificially recombinant libraries of TCRs displayed on phage or yeast, as vectors for discovery of antigen‐specific clones [61, 62, 63]. Together, these origins incorporate additional, non‐natural sequence/structural diversity to the characterized TCR space.

## 2 TCR Structure Resources

### 2.1 Experimental Structure Databases

**FIGURE 2:**
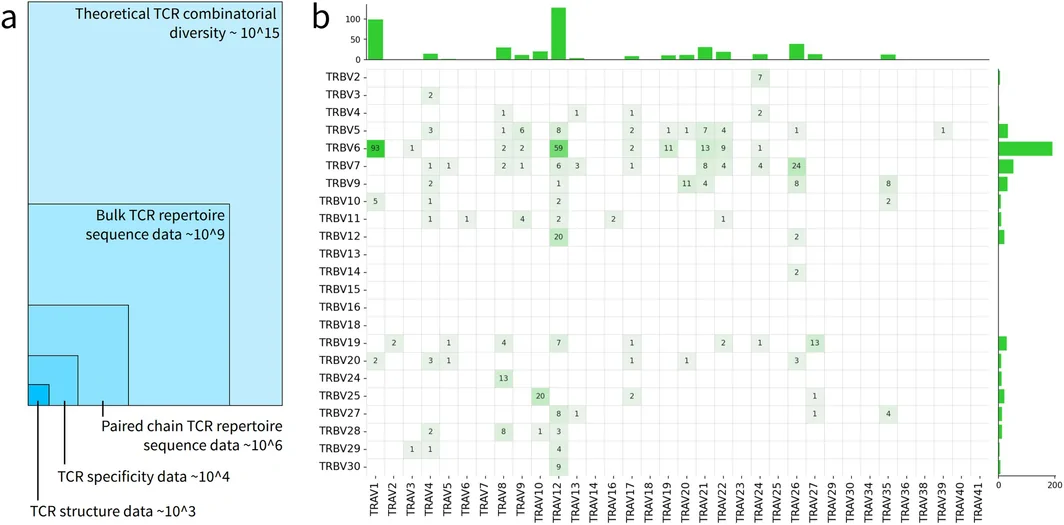
(a) Diagrammatic representation of TCR data modalities and quantities of available data. (b) Data biases in the publicly available set of TCR structures used to train TCRBuilder2+ [23]. TRAV and TRBV gene sub‐groups on the *x*‐ and *y*‐axis, respectively.

Reflective of the increasing interest in TCR structures, databases such as STCRDab [24] and TCR3d [64], which curate TCR and TCRm antibody data deposited in the PDB [65], have been published. As of July 2026 a total of 781 PDB entries had been consolidated in these repositories (Table 1). Of these, 577 contain a TCR bound to an MHC or MHC‐like complex, the remaining 204 structures are *apo*, that is, unbound TCRs. The curation of this data has enabled systematic and comparative analyses of TCR structure data, however, the number of depositions represents an order of magnitude fewer data than are available for antibody or TCR sequences (Figure 2a) and imposes a particular constraint on machine learning pipelines, which require large quantities of data. There are some fundamental technical limitations behind this disparity. For example, cryogenic electron microscopy (cryo‐EM) as a technique now contributes more antibody structural data to the community each year than X‐ray crystallography; roughly 38% of the 10,700 structures in Structural Antibody Database (SAbDab) were solved by cryo‐EM, as of April 2026 [68]. The routine application of cryo‐EM to determine the structure of TCR Fvs may be hindered by the existence of established crystallography protocols, as well as by the poor stability of complexes during sample preparation due to low‐affinity binding [69]. Furthermore, the relatively small size of the soluble components of the TCR:pMHC complex may present challenges for cryo‐EM analysis [69]. As a result, despite the recent success of applying cryo‐EM to probe the larger TCR–CD3 complex [70], X‐ray crystallography remains the dominant source of TCR Fv structures. In the 6 months from November 2025 to May 2026, 29 new TCR structures were deposited in the PDB, though only 15 of these emerged from independent studies [64]. Of these, 18 structures were resolved with X‐ray crystallography, and, indicative of growing interest, 10 of the resolved structures are of gamma‐delta TCRs.

**TABLE 1:** Table of resources relevant for TCR structure analysis and prediction.

Resource name	Description (number of datapoints)
STCRDab [22]	TCR structures (634 TCR and TCR:pMHC)
TCR3d [59]	TCR & antigen structures (781 TCR and 1752 MHC)
FTCRDab [66]	TCR molecular dynamics (MD) trajectories (280 apo TCR)
Observed TCR Space (OTS) [67]	Paired‐chain TCR sequences (2.3M)
OTS Predictons [23]	Predicted TCR structures (1.5M)
STCRpy [68]	Analysis and processing of TCR and TCR:pMHC structures
CV3‐Fv [66]	Protocol for fast TCR MD simulations
TCRBuilder2+ [23, 32]	High‐throughput *apo* TCR structure predictor
TCRmodel2 [34], TFold‐TCR [35], TCRdock [19]	TCR:pMHC complex predictors

*Note:* Data resources in top half of table, software tools in bottom half of the table. Number of datapoints as of May 2026. List of structure predictors reduced to TCR‐specific deep learning methods.

In part due to many studies leading to multiple structure depositions each, a key consideration is the relative lack of diversity across solved structures. We recently quantified this data bias in a study on publicly available data as well as 202 privately held structures contributed by collaborators at Immunocore [25]. Parallel analysis of these datasets revealed common strong biases towards a small number of genes and gene sub‐groups and that the joint distribution over TRAV‐TRBV gene pairs is sparsely sampled. The majority of potential V‐gene pairs that could arise in an immune repertoire remain unsampled or have only been structurally characterized once (Figure 2b). In addition to sparse sampling, the joint distribution was disproportionately weighted towards a small subset of gene pairs; for example, TRAV12‐2 paired with TRBV6‐5, and TRAV1‐1 with TRBV6‐1, account for 79 and 59 structures respectively, 15.7% of the combined data. These biases indicate that, to date, a narrow pool of similar TCR structures has been solved, likely in conjunction with research into specific diseases and targets. In summary, while we have rich understanding of TCR structures in particular contexts, the overall landscape of solved TCR structures remains unexplored.

### 2.2 Software for Handling and Processing TCR Structures

While experimentally resolved TCR structures remain a sparse resource, the release of accurate in‐silico structure predictors has enabled the generation of large quantities of predicted TCR structure data. The increased availability of data emphasized the bottleneck imposed by the relative complexity of handling and analyzing TCR and TCR:pMHC structures. While immunoglobulin‐specific software packages have existed for over a decade to enable sequence‐based TCR analysis [71, 72], we identified recently that an equivalent suite for the annotation and processing of TCR structure data was lacking [24, 64]. We developed STCRpy [66], a python package to automate the processing and characterization of solved and predicted TCR:pMHC complexes or *apo* TCRs, to address this bottleneck.

STCRpy parses TCR:pMHC complexes from local PDB or MMCIF files, or retrieves structures directly from STCRDab [24] or the PDB [65]. Protein chains are first annotated using a modified version of ANARCI which is able to classify MHC and MHC‐like sequences as well as immune receptors [72]. All TCRs are then numbered and annotated by region according to the IMGT system/definitions [44]. TCRs and antigens are paired by distance into separate “TCR” objects, accounting for multiple TCR:pMHC copies per PDB file.

One bespoke processing step integrated in STCRpy is the calculation of the relative orientation of the TCR to the pMHC, which is a key property linked to the ability of cell‐surface TCRs to trigger downstream signaling [67]. This is particularly useful as many general protein–protein complex prediction software packages generate poses of TCRs that fall outside the “canonical” angles consistent TCR co‐receptor signaling. There are contexts in which signaling, and by extension the TCR scanning angle, is of lesser importance, for instance when designing soluble TCRm binders, which do not (directly) stimulate an intracellular signaling pathway [57].

To facilitate the generation of structure‐based machine learning pipelines, STCRpy can parse both *apo* and bound TCR complexes into datasets of graphs using the popular pytorch‐geometric and pytorch deep‐learning libraries [73, 74]. The generated graphs are compatible with the existing pytorch‐geometric suite of graph neural network architectures and can be integrated flexibly into training paradigms of bespoke neural networks. Finally, STCRpy profiles interactions between the TCR and the antigen with PLIP [75], thereby characterizing hydrogen bonds, salt bridges, and hydrophobic or aromatic interactions. This simplifies the mechanistic interpretation of the TCR:pMHC interface, helping to highlight crucial interactions.

## 3 Insights From TCR Structure Analysis

### 3.1 Principles of TCR:Antigen Recognition

Crystal structures of TCR:pMHC complexes have shown that the hyper‐variable CDR3 loops make primary contact with the peptide, while also engaging the MHC, and that germline‐encoded CDR1 and CDR2 loops contact both the MHC and the peptide, but often to a lesser degree [15, 23]. This suggests that peptide and TCR CDR3 complementarity is the key driver of specificity, though sufficient contact to the MHC is requisite for binding [15]. Contact between the peptide and the CDR3 loops is facilitated by the canonical docking angle observed for natural TCRs, which is imposed by the signaling geometry of the TCR‐CD3 complex [67]. Specifically, productive signaling requires co‐localization of the TCR‐CD3 complex with the CD4 or CD8 co‐receptors, which enforces a canonical, diagonal docking orientation of the TCR over the pMHC [67]. Corroborating this theory of signaling, non‐canonical TCRs, which bind with so‐called “reverse polarity,” have been observed not to stimulate down‐stream signaling [76]. Recently, when developing STCRpy, we characterized the distribution of TCR:pMHC Class I pose angles, revealing an approximately normal distribution of angles centered about 67.9° ± 14.2°, when calculated using the “centre of mass” angle determination method [66].

Our recent analysis of 60 representative TCR:pMHC structures revealed the broad interface of TCR:pMHC complexes relative to antibody: antigen complex structures, enabled by the relatively flat topology of pMHC antigen [57]. Furthermore, our analysis of nonamer peptides presented by Class I MHC revealed that at least five, but more commonly six or seven peptide residues are buried by the TCR at the interface, with four to five residues directly interacting with TCR on average. TCRs reliably exploit energy hotspots on the peptide surface, and free‐energy calculations confirmed that the peptide and CDR3 loops make the largest relative contributions to energetic changes [57]. The broad interface and observation of interactions across several residues may partially explain the observed cross‐reactivity of TCRs to similar peptides, and, coupled with the relatively low affinity observed for TCR:pMHC complexes, is indicative of a highly sensitive, distributed TCR:pMHC interface [77].

### 3.2 Engagement of pMHC Antigen by TCR‐Mimics Differs From TCRs

The high sensitivity and relatively low binding affinity (1–100 μM) of natural TCRs has motivated efforts to engineer high‐affinity (100–1000 nM) antibody Fv regions which bind to pMHC, so‐called TCR‐mimetic (TCRm) antibodies [57]. TCRms are not subject to the same geometric signaling constraints as TCRs, and our recent comparative analysis of 10 available TCRm antibody:pMHC structures revealed that TCRm antibodies exhibit different binding patterns, including frequent reverse polarity binding modes [57]. Specifically, we profiled the complete set of available TCRm antibody:pMHC crystal structures against representative TCR:pMHC complexes, quantifying buried surface area, CDR loop contributions, docking geometry, and binding energetics. Despite the genetic analogy between antibody VH/VL chains and TCR Vβ/Vα chains, their CDR loops were found to play different roles in pMHC recognition. In particular, the CDR2L of TCRms is not able to recapitulate the antigen contact commonly made by the CDR2α, likely reflective of the infrequent antigen contact made by the CDR2L of antibodies against general antigen.

Furthermore, TCRm antibodies exhibit a systematic bias towards greater MHC surface burial at the expense of peptide contacts, covering fewer peptide residues on average and deriving a lower fraction of their binding free energy from the peptide in all three pMHC contexts examined by molecular dynamics. Specifically, for nonamer peptides presented by MHC Class I, burial of eight peptide residues is not uncommon across TCRs (17.2%) but has not yet been observed in any TCRms. Furthermore, only a single nonamer‐pMHC Class I‐binding TCRm antibody (1/7, 14.3%) achieves burial of seven peptide residues, whereas 13/29 (44.8%) of the TCRs examined attain this level of peptide coverage. Instead, TCRm antibodies appear to achieve higher antigen affinity than TCRs through hotspot interactions with MHC residues. Binding hotspots on the MHC rather than the peptide likely increase the risk of cross‐reactivity, as has been reported for the ESK1 TCRm [78]. That said, the degree of TCR‐likeness varies substantially across TCRm antibodies and appears clinically relevant: the two TCRms that have advanced to human clinical trials engage pMHC with considerably more peptide‐focused burial profiles than ESK1, a TCRm antibody with documented off‐targets that has not progressed through preclinical development [57]. In summary, analyzing antibody and TCR structural resources in parallel can reveal details of where productive TCRs, whether through evolution or somatic selection, land in a niche that permits systematic peptide‐sensitive recognition, and these lessons could inspire the development of new pMHC‐binding modalities.

### 3.3 CDR Conformational Changes Upon Binding

One suggested mechanism for the high affinity and target‐specificity of antibodies is the reduction of the entropic cost of binding through the rigidification of the CDR loops, potentially as a consequence of somatic hypermutation [79]. By contrast, TCRs, which do not undergo somatic hypermutation, have been observed to adopt multiple conformations, further diversifying the TCR‐pMHC interface [80]. Structural and molecular dynamics (MD) studies have shown that TCR CDR loops can adopt multiple conformations and that binding can induce conformational changes in both the receptor and the peptide [15, 80, 81]. Our recent work analyzed 391 structures, comprising 22 TCRs, 19 MHC alleles, and 79 peptide structures in both unbound (*apo*) and bound (*holo*) conformations [80]. The analysis revealed that 54% of TCRs exhibit changes in their CDR loop conformations with backbone deviations between 1 and 5 Å when comparing the *apo* and *holo* structures. Conformational changes were greatest for the CDR3 loops of both the α and the β chain, likely due to greater average loop length and their higher sequence diversity. Furthermore, analysis of the peptide conformations of the antigen revealed statistically significant shifts in peptide conformation upon TCR engagement. Independent studies which crystallized both *apo* and *holo* pMHC structures identified that conformational changes in the antigen affect TCR binding efficacy in vitro [81]. Together, these results provide insights into the dynamic nature of TCR‐pMHC engagement, and highlight the importance of considering multiple conformations of TCRs and pMHC when conducting in silico analysis of these molecules and their interfaces. In particular, the observed flexibility implies that the TCR conformation relevant for antigen recognition may differ from that of the unbound receptor, and that conformational heterogeneity and induced fit should be accounted for when analyzing and predicting TCR structures.

In the absence of good experimental “ground truth” conformational data with which to explore CDR flexibility, MD can offer a perspective into protein dynamics at atomic resolution, and this technique is gaining traction in the study of TCR and antibody flexibility [82, 83, 84, 85]. All‐atom MD is particularly well‐suited for capturing a range of molecular interactions, however, the high computational cost limits long‐timescale sampling and the number of proteins that can be simulated [82]. Coarse‐grained (CG) simulations offer a more scalable alternative, as they reduce the number of particles in the system and thereby increase sampling efficiency. Recently, we developed a protocol for CG MD of Fv regions of antibodies and TCRs using the CG protein dynamics model CALVADOS 3, which was designed for multi‐domain proteins [82, 86, 87]. Our protocol design was motivated by the observation that, when classifying the entire CDR loop as defined by IMGT numbering as an “unfolded” region, CALVADOS 3 simulations of the Fv tended to overestimate the flexibility of all CDRs. To capture the intra‐ and inter‐CDR interactions in our simulations more accurately, we applied additional constraints to the loops using the interactions profiled from the input structures, and named this setup CALVADOS 3‐Fv (CV3‐Fv) [82]. Having verified the consistency of our CG protocol with all‐atom simulations, we applied it to 280 TCR Fv structures from STCRDab [24], generating the Flexible TCR Database (FTCRDab), the first such repository for TCR MD data. Using our CG protocol, each TCR Fv flexibility profile takes only two hours on a standard CPU, an order of magnitude less time than all‐atom simulations [82]. The low computational resource requirements mean the FTCRDab can easily be extended as new solved TCR structures emerge, and can be applied to other datasets using moderate compute budgets. Future work could even extend these simulations to predicted TCR structures as well as crystal structure data, enabling the estimation of conformational variance of TCR Fv's directly from sequence via the in silico structure prediction. Incorporating TCR structure predictions into MD simulations, as we have previously demonstrated for antibodies [82], would represent an order of magnitude increase in available TCR flexibility and conformation data.

## 4 TCR Structure Prediction

### 4.1 Apo TCR Fv Structure Prediction

#### 4.1.1 Template‐Based Homology Modeling Enabled the First In Silico TCR Structure Predictions

The cumbersome process for obtaining experimentally determined structures, coupled with recent advances in protein structure prediction, has made computational modeling a desirable approach for studying TCRs structurally. Early approaches to TCR 3D structure prediction relied on homology modeling, in which the structure of a query sequence is inferred by alignment to a structurally characterized template. MODELER [88], ROSETTA [89], and SWISS‐MODEL [90] are some of the best known homology models for general proteins. However, homology modeling is limited in cases where no template structure can be found, and is dependent on the alignment of the query to the template structure. Furthermore, TCRs and pMHC antigen have regions which are highly conserved both in structure and in sequence, while the diversity of the CDR3 loops and peptide, which are predominantly the regions of interest, means their structures vary in key regions despite high sequence identity. To account for these idiosyncrasies, TCR‐specific homology tools were developed, including TCRBuilder [91], TCRmodel [92], and LYRA [93]. Comparisons of these homology models revealed that template‐based modeling achieves < 1.5 Å RMSD (root‐mean‐square deviation) for the germline‐encoded and framework regions but that the CDR3 loops often require free modeling when no template can be found, and that these loops are modeled with median RMSD > 2 Å regardless of the modeling method [45].

#### 4.1.2 AlphaFold2 and Derivative Models Offer Improvements in CDR3 Loop Prediction

Deep‐learning methods offer a compelling alternative to homology modeling by seeking to optimize neural network parameters to approximate the mapping from protein sequence to structure. The first iteration of the AlphaFold architecture, which was based on convolutional neural networks (CNNs), dominated the 13th round of the Critical Assessment of protein Structure Prediction (CASP13) methods [94]. AlphaFold2, the subsequent Transformer‐based architecture released in 2021 [26], is widely considered to have solved protein structure prediction for the majority of natural proteins. The impact of AlphaFold2 on structural bioinformatics can hardly be overstated and forms the basis of most protein structure prediction algorithms today [95]. As well as predicting the atomic coordinates of a protein structure, AlphaFold2 also produces a quality metrics, such as the predicted local‐distance‐difference‐test (pLDDT), which can be interpreted as uncertainty estimates of the predicted structure [26].

Multiple extensions and adaptations of AlphaFold2 have been published since the original paper and code were made available. These include the extension to multimeric proteins, dubbed AlphaFold‐Multimer [96], as well as reproductions such as OpenFold [28] and alternatives such as RosettaFold [97]. Improving protein structure prediction, particularly of complexes, remains an active area of research and the release of AlphaFold3 [27] heralded the latest generation of diffusion‐based models, including Chai [33] and Boltz [31].

Motivated by the observation that, similar to homology models, AlphaFold2‐style prediction models struggle to accurately predict the CDR3 loops of TCRs and antibodies [34], bespoke model versions to predict the Fv regions of immune proteins were developed. We developed a fast, high‐throughput TCR‐specific structure prediction tool as part of the ImmuneBuilder model suite [34]. The development of ImmuneBuilder [34] recognized that, in contrast to general proteins whose sequence diversity is subject to evolutionary pressure such that the structure and function of the protein stays the same, immunoglobulin sequences are the product of somatic recombination and have evolved to be structurally diverse and specific to different antigens. ImmuneBuilder comprises a suite of three models trained on antibody, nanobody, or TCR structures. Each of these models consists of an ensemble of structure modules which accept one‐hot encodings of the immunoglobulin sequence as input. The first version of ImmuneBuilder trained on TCRs was called “TCRBuilder2” [34], and the second version, which was trained on a larger dataset, was called “TCRBuilder2+” [25]. While the original TCRBuilder2 model followed from ABodyBuilder2 and selected the ensemble of trained models based on only the VDJ‐recombined CDR3H's validation set accuracy, for TCRBuilder2+ we adapted the ensemble selection to include the accuracy over both chains, better suited to the TCR paratope, which is distributed across both the CDR3 loops. The performance of TCRBuilder2+ improved for genes better sampled in the new training set, and TCRBuilder2 and TCRBuilder2+ both achieved comparable overall accuracy to Alphafold‐Multimer at a fraction of the computational cost by ablating the MSA generation and processing steps [25].

Other TCR structure prediction tools derived from AlphaFold2 are TCRmodel2 [36] and TFold‐TCR [37], both of which adapt the AlphaFold modeling pipeline and can make TCR:pMHC complex predictions as well as *apo* TCR predictions. We expand on these methods in Section 4.2.5 on TCR:pMHC complex modeling strategies. An independent benchmark compared the prediction accuracies of *apo* TCR structure prediction models, including the homology model LYRA [93]; TCR‐specific deep learning models TCRmodel2 [36], TCRBuilder2 [34], TCRBuilder2+ [25], and tFold‐TCR [37]; and the general protein structure predictors AlphaFold2 [26], and AlphaFold3 [27]. Broadly, these models perform similarly, including LYRA, suggesting that the gains made by switching to deep learning methods are, on the whole, moderate [98]. Deep learning models do offer performance gains in the CDR3 regions compared to homology modeling, indicating that some generalizable properties learnt during model training transfer to unseen CDR3 loops. The current average RMSD of deep learning models lies between 1.5 and 2.5 Å for CDR3β loop and between 1.5 and 3.0 Å for the CDR3α loop [25, 98]. Nonetheless, the high variance in CDR3 loop prediction accuracy suggests this problem is far from solved, with errors ranging beyond 5 Å for some test cases [98].

#### 4.1.3 TCR Structure Predictions Are Indicative of Equivalent CDR3α and CDR3β Structure Diversity

Evaluation of *apo* TCR structure prediction methods revealed that both TCR‐specific and general protein structure predictors achieve comparable accuracy when modeling the VJ‐recombined CDR3α and the VDJ‐recombined CDR3β loops [25, 98]. Further examination of the landscape of solved TCR structures indicated that the VJ‐recombined α chain junction is at least as structurally diverse as the VDJ‐recombined β chain junction, with CDR3α loop structures showing little tendency to cluster into canonical forms [25]. Earlier efforts to assign CDR3α loops to canonical structural classes provided corroborating evidence: sequence‐based cluster assignment is less consistent for CDR3α than for CDR1α and CDR2α [45], and canonical class‐based structure inference performs less well for CDR3α than for CDR3β [93]. Quantitatively, while 76.8% of CDR1α loops (43/56) and 72.0% of CDR2α loops (36/50) were successfully assigned to a canonical cluster, the corresponding figure for CDR3α was only 22.4% (24/107) [45]. This stands in marked contrast to the pattern observed in B‐cell receptors and antibodies, where VJ‐recombined CDR3L loops predominantly adopt canonical conformations despite the analogous diversification mechanism, while VDJ‐recombined CDR3H loops are considerably more structurally variable [34, 45, 99].

A plausible functional explanation for this divergence lies in the different roles played by the VJ‐recombined loop in each receptor type. In antibodies, the CDR3L typically contributes indirectly to binding, with antigen specificity primarily driven by the CDR3H [100], whereas available evidence suggests that the TCR CDR3α and CDR3β contribute more equally to pMHC recognition [80]. In the antibody context, this asymmetry is supported by findings that heavy chain clonotype predicts binding specificity whereas light chain clonotype does not [101], and by metadynamics simulations demonstrating that CDR3Hs preferentially sample conformations in regions of space unoccupied by the CDR3L loop, but not the reverse [83].

In addition, the CDR3α loop length distribution is similar to that of CDR3β, whereas the CDR3L is shorter than the CDR3H in antibodies [57]. Given that both TCR CDR3 loops frequently contact the peptide, it is consistent with evolutionary logic that selective pressure would have acted to increase CDR3α structural diversity in parallel with that of CDR3β, thereby broadening peptide sensitivity.

This interpretation is supported by the combinatorial diversity accessible to the α chain, which exceeds that of the β chain, thereby potentially compensating for the absence of a D gene segment [44]. The larger number of TRAJ genes (61) relative to TRBJ (14) or the immunoglobulin light chain joining genes IGLJ and IGKJ (11 and 5 respectively) is the primary driver of this difference. As a result, α chain combinatorial and structural diversity considerably exceeds that of the antibody light chain, which remains less variable despite the additional diversification conferred by somatic hypermutation. Taken together, this elevated diversity in the α chain VJ junction, combined with the relative scarcity of available structure data, likely accounts for the comparable prediction errors observed for CDR3α and CDR3β across current models. Nevertheless, the strong structural coherence observed within TRAV‐TRAJ gene pairs, together with the prediction improvements seen for better‐sampled genes, suggests that targeted expansion of our databases of solved TCR structures could yield meaningful gains in prediction accuracy [25].

### 4.2 TCR:pMHC Complex Structure Prediction

#### 4.2.1 Docking Algorithms

Accurately predicting protein interfaces remains challenging, particularly for receptors such as TCRs with diverse antigens and paratopes. Prior to the development of deep learning co‐folding models, docking algorithms were the only option for predicting the relative poses of two proteins, and in some benchmarks, these algorithms remain competitive with deep learning models for immune receptors [102].

TCR‐specific docking protocols seek to address the unique properties of TCR and pMHC, such as diversity and conformational flexibility of the CDR loops. TCRFlexDock [103] perturbs and refines the CDR3 loops of the TCR and docks to the pMHC in two phases using RosettaDock [104]. SwifTCR improves the efficiency of classical docking algorithms by reducing the search space for poses [105], and TCRpMHCmodels [106] is a homology‐based approach, which predicts the TCR and pMHC structure separately before assembling them with MODELLER [88]. A comparison of general docking algorithms indicated that the method HADDOCK performs best for TCR:pMHC complex prediction [107]. HADDOCK is designed to incorporate constraints from experimental data or prior knowledge of the interface by permitting the definition of “active” and “passive” residues which form the interface of the docked structures [108, 109]. Furthermore, after an initial, biased rigid‐docking iteration, docked poses are flexibly refined, allowing for small changes in conformation at the interface.

#### 4.2.2 TCR‐Specific Scoring Functions for Docked TCR:pMHC Candidates

Unlike deep‐learning complex predictors (Section 4.2.5), docking methods do not have a data‐derived prior for the final complex. Rather, they produce many highly diverse candidates (often in the hundreds) and rank these according to some proxy for affinity. These scoring functions often perform poorly when retrieving the best complex compared to a ground truth benchmark [110]. Specifically, in docking case studies, we found that the docking scores of the best ranked dock correlate with interface RMSD (iRMSD), but that only 31.1% (14/45) of the best docks by iRMSD also had the best docking score [25]. An additional challenge faced by docking scores is that, as they are commonly derived from energetic enthalpy terms only, they cannot be compared across different TCR and pMHC pairs. The unreliability of general pose ranking functions in this domain limits the practical applicability of docking for investigating TCR:pMHC complementarity and motivates the development of TCR‐specific scoring functions.

In order to better evaluate and rank the TCR:pMHC complexes generated during the docking simulations, we recently defined a TCR‐specific scoring function exploiting the known canonical orientation of TCRs above the pMHC (see Section 3.1) [66]. We characterized the TCR:pMHC complexes by geometric features which define the TCR's pose relative to the MHC: the scanning angle θ, the pitch angle ψ, and the z‐component of the TCR's centre of mass. Calculating these metrics for all the canonical pose α βTCR to Class I pMHC complexes in STCRDab [24] enabled us to fit simple parametric distributions to the data via maximum likelihood estimation. We then calculated the likelihood of any query TCR:pMHC complex prediction arising from these feature distributions, and convert these into a cumulative score, such that the highest scores indicate the most “TCR‐like” docking predictions.

Evaluation of this TCR:pMHC docking scoring function on HADDOCK case studies demonstrated that AUC of the most accurate predictions improved to 0.843 from 0.631 compared to using HADDOCK's internal docking score alone [66]. Considering both the docking score and TCR:pMHC geometry score yielded slightly worse AUC of 0.836 across all the complex predictions, but improved the recall of accurate predictions in the top 50 scored complexes. Evaluating the TCR:pMHC predictions made by AlphaFold3 [27] yielded a positive correlation (PCC of 0.641 with *p* of 0.0459 < 0.05) between the interface RMSD and the TCR:pMHC geometry score, implying that the scoring function retains utility even when the modeling method has a data‐driven prior for TCR‐like complex poses.

#### 4.2.3 Docking Apo TCR Structure Predictions Into Antigen Structures Reveals a Prediction Accuracy Threshold for the CDR3 Loops

Hybrid protocols have been used whereby TCR structure predictions generated with deep learning models are docked into known or predicted antigen structures. In recent work, we predicted TCR structures from sequences using both TCRBuilder2+ [25] and Alphafold‐Multimer [96] and docked these predicted structures to antigen crystal structures with HADDOCK2.4 [108]. To ensure the paratope and epitope were correctly identified, we devised a protocol for TCR:pMHC complex docking with HADDOCK whereby the residues of the six TCR CDR loops and the peptide residues are defined as “actively” constrained residues in the simulation; additional unburied MHC residues within 6 Å of the peptide were defined as “passively” constrained residues. The resulting 200 docked poses per TCR structure prediction were ranked using both HADDOCK's energy term and the bespoke, data‐derived likelihood score [66, 108].

Analysis of the prediction errors and ability to retrieve accurate TCR:pMHC complex structure predictions revealed that accurate prediction of both the CDR3α and the CDR3β loops is required to enable accurate docking to pMHC [25]. A specific threshold which emerged from these case studies was that a combined RMSD over the CDR3α and CDR3β < 3 Å of the *apo* TCR prediction consistently enabled the retrieval of a docked candidate complex with an iRMSD < 4 Å. For comparison, re‐docks of TCR crystal structures indicate that the best attainable iRMSD lies between 3 and 3.5 Å [25]. Further investigation of the predictive errors revealed that no consistent accuracy threshold could be identified when the CDR3α or CDR3β prediction errors were considered separately, reinforcing the notion of a joint interface to the antigen. Though it was possible to retrieve TCR:pMHC poses with iRMSD < 4 Å for nearly half of the TCRs investigated, frequently these were not the docks with the best HADDOCK docking score, reiterating the limitations of general scoring functions.

In addition to docking scores, most *apo* TCR structure prediction tools produce confidence scores with which multiple predicted conformations of the TCR can be ranked. However, our case studies revealed that the best‐ranked *apo* TCR structure prediction does not consistently yield the most accurate docked complex [25]. This implies that including multiple conformations of TCRs during docking improves the accuracy of the best predicted complex, although retrieving this hypothetical “best dock” remains challenging without effective scoring functions or a so‐called “oracle” ground‐truth complex structure against which the candidate poses can be evaluated. We hypothesize that the inclusion of multiple conformations of *apo* TCRs combinatorially expands the search space for poses, thereby increasing the chances of retrieving a good docked pose, especially in the context of induced fit of the CDR loops upon binding [80, 91]. Including multiple conformations in docking studies has been shown to improve the retrieval of accurate structures for antibody: antigen complexes [102].

#### 4.2.4 Hybrid Docking Protocols Using Apo TCR Structure Predictions Can Generate Structural Hypotheses for Experimental Data

Advances in docking and complex prediction methods have enabled the development of computational structural hypotheses for TCR engagement of antigen, which can yield insights for TCR affinity and specificity engineering. For instance, recent work identified a TCR that specifically recognizes both a KRAS G12V and G12C neoantigen presented by the prevalent human leukocyte antigen HLA‐A*03:01 allele [111]. Experimental assays and in vivo studies demonstrated that combining lymphodepleting chemotherapy with T cell‐based therapies, including TCR‐T cells, tumor‐infiltrating lymphocytes, or a T‐cell engager antibody, enhanced antigen‐specific tumor killing across multiple cancer types and antigens when using this newly discovered TCR [111]. We computationally investigated the structural basis of specific recognition of both the KRAS G12V and G12C peptides by this TCR and performed in silico modeling of the TCR‐peptide‐HLA complexes, with a view to use the resulting models to inform potential mutations that could further enhance the TCR.

Using *apo* TCR predictions docked into structures of the KRAS antigens, and then evaluating the resulting candidate complexes with TCR geometry‐derived scoring functions, we developed a structural hypothesis for specific TCR engagement. This structural hypothesis supported a unified mode of recognition of KRAS G12V and G12C by the identified TCR, consistent with the experimental findings of functional recognition of the G12V and G12C mutations, but not the wild‐type (WT). Our characterization of this interface suggests that a hydrophobic region generated by the CDR3α and CDR3β facilitates recognition of the antigen, consistent with independent results showing that the recognition of KRAS G12V relies on hydrophobic interactions [112, 113]. Nonetheless, in the face of modeling inaccuracies that remain challenging to evaluate, we considered the docked structure prediction as a physically plausible hypothesis; as with all computational predictions, it remains possible that experimental resolution of the crystal structure of the complex would reveal an alternative mode of engagement.

Despite the remaining hurdles and difficulties, the accelerated progress in the field of in silico protein complex prediction has enabled the generation of rational hypotheses for TCR:pMHC complexes at a fraction of the cost of experimental methods, thereby improving the accessibility of structural rationalization of experimental results. Circumventing the need to dock *apo* structures, recent developments in general protein complex prediction use co‐folding methods, where the entire complex is predicted from directly from sequence [27, 31, 33].

#### 4.2.5 Deep Learning Models That Jointly Predict the TCR and pMHC Complex

Distinct from docking TCRs to pMHC molecules, deep learning methods have been developed which predict the entire TCR:pMHC complex directly from sequence. Modeling directly from sequence is advantageous when the structure of both the TCR and the pMHC is unknown. AlphaFold2 [26], and its extension to complexes, AlphaFold‐Multimer [96], were the first models to attain prediction accuracies which improved on bespoke TCR docking methods, and TCR‐specific derivative models of AlphaFold‐Multimer have been developed to improve the modeling accuracy further [98].

TCRmodel2 [36] adapts the AlphaFold‐Multimer pipeline in three ways. Firstly, the sequence database used to generate multiple sequence alignments (MSA) is reduced to TCR and MHC sequences only, thereby accelerating the MSA generation step. Secondly, the search for TCR and pMHC structure templates was adapted. For TCRs, the structure search uses the TCR sequence rather than the MSA, which improved the retrieval of TCR templates. To improve pMHC template searches, pMHC structure templates were retrieved from TCR3d [64], and candidates with length‐matched peptides were first ranked by MHC sequence similarity, and then by peptide sequence similarity if multiple MHC candidates are found. Thirdly, the confidence score was altered to emphasize the interface between the TCR and pMHC, rather than the interfaces between all chains. These changes improved the accuracy of TCR:pMHC complex predictions compared to AlphaFold2, as well as reducing the computational cost of generating predictions [36].

Similarly, TCRdock incorporates domain‐specific structural templates into the AlphaFold2 pipeline by generating candidate docked poses from existing, known TCR:pMHC poses [21]. For each prediction target, separate templates are selected for the pMHC, TCRα, and TCRβ chains by sequence identity, and these are combined with a set of representative docking geometries derived by clustering known TCR:pMHC structures to construct 12 input templates for AlphaFold2. To further improve performance on TCR:pMHC complexes, the model parameters were fine‐tuned on a training set of 93 human TCR:pMHC structures. Benchmarking suggests that these changes improve TCR:pMHC complex prediction, but that the predictions are not consistently more accurate than the best, “oracle” template dock that was provided as input [21].

TFold‐TCR [37] fine‐tunes the protein language model ESM2 [114] both on protein–protein‐interactions as well as TCR and pMHC sequence data to generate sequence embeddings, and then retrains two separate, smaller versions of AlphaFold2's “Evoformer” and “Structure Module” to produce both TCR and pMHC complex predictions. The resulting predictions and embeddings are fed into another embedding module, which uses cross‐attention to better capture interactions between the TCR, peptide and MHC chains. Finally, the resulting embeddings are passed through another small version of the AlphaFold2 architecture, trained exclusively on TCR:pMHC structures. Using ESM2 to embed the sequences circumvents the need to generate the MSA or the MSA embeddings, making TFold‐TCR faster compared to other AlphaFold‐derived models [37].

AlphaFold2 [26], Alphafold‐Multimer [96], and their derivative models are regression models, which means they are optimized to approximate the function that maps from sequence inputs to structure outputs. AlphaFold3 [27] moved away from this paradigm by defining a generative structure prediction model based on diffusion models [115]. Under this framework, a structure prediction from sequence is generated by iteratively updating randomly initialized, noisy coordinates, which enables a more flexible definition of the model inputs. Formally, the diffusion model defines a mapping from a noisy starting distribution (p_0_) to a final, “clean” distribution (p_1_) over the atomic coordinates, x, conditioned on the sequence, s: M: p_0_(x|s) → p_1_(x|s). This enables structure predictions to be sampled many times by repeatedly drawing random coordinates from p_0_, which is chosen to be a normal distribution, and updating these with the trained neural network until a final prediction has been made, which is a generated sample from the learnt data distribution p_1_. For a more granular exploration of generative co‐folding methods, we refer to related literature on diffusion models [115, 116], the AlphaFold3 implementation details [27], as well as recent work characterizing the predictions made by diffusion‐based co‐folding models [117, 118].

Co‐folding models such as AlphaFold3 enable broader definitions of inputs as well as the possibility of guiding and steering the structure generation towards desirable properties. Guidance and steering are techniques used to shift the generated samples of diffusion models towards some other distribution, often referred to as “tilting” the distribution [119, 120, 121]. One of the appeals of these methods is that many can be applied to a pre‐trained diffusion model, without needing to retrain or fine‐tune the parameters [121, 122]. Similar to AlphaFold2, the publication of AlphaFold3 led to the release of a plethora of reimplementations, including OpenFold3 [29], Boltz [31], Chai [33], and Protenix [30]. No TCR‐specific versions of AlphaFold3‐like diffusion models have been released to date. This could be reflective of the relatively recent release of AlphaFold3; however, it could also reflect the limited improvements in accuracy that were observed after retraining or fine‐tuning AlphaFold2 on TCR data [98].

Recently, an independent benchmark compared existing TCR:pMHC complex structure predictors, including the homology and docking model TCRpMHCmodels [106], TCR‐specific AlphaFold2‐like models, TCRmodel2 [36], TCRdock [21], and tFold‐TCR [37], and the general protein predictors, AlphaFold2 [26, 96] and AlphaFold3 [27]. All deep learning predictors outperformed the docking method TCRpMHCmodels [106], differentiating TCR:pMHC complex prediction from *apo* TCR structure prediction, where the improvements attained by deep learning models were noticeable but less pronounced. Among these, tFold‐TCR is marginally weaker, though differences on the standard interface metrics iRMSD, the fraction of recovered natural contacts (fnat), and DockQ (defined as the mean average of the fnat, scaled iRMSD, and scaled ligand RMSD scores [123]) remain within the margin of error across methods [98]. Complex structure predictors are approaching accuracies at which they could reasonably be used to characterize TCR:pMHC interfaces, with the median iRMSD scores below 3 Å for all deep‐learning models [98]. Nonetheless, the low median fnat scores (below 50%) are revealing of how far TCR:pMHC complex structure prediction methods have yet to come before atomic‐level interactions can consistently be recapitulated.

### 4.3 Inferring TCR Specificity From TCR:pMHC Complex Predictions

TCR specificity prediction is considered a “holy grail” of computational immunology research, and tens of articles have been published in the last five years applying machine learning methods to this task for example, [124, 125, 126, 127, 128, 129, 130, 131, 132, 133, 134, 135, 136, 137, 138, 139, 140, 141, 142, 143, 144, 145, 146, 147, 148, 149]. Due to the volume of TCR specificity research being published, and the success of competition‐style assessments such as CASP for protein structure prediction [150], the IMMREP competition series was launched, of which three iterations have been completed (2022 [18], 2023 [17], and 2025 [39]). During the competition researchers are invited to contribute models and predictions which are evaluated against a withheld test set, which facilitates a direct comparison of methods and provides an overview of the current state‐of‐the‐art and general limitations. The first two IMMREP reports highlighted the continued difficulty of generalizing to unseen epitopes, the importance of paired chain TCR data, and issues surrounding synthetic negative data generation [17, 18]. Recent work estimates that approximately 1000‐times more data is required to enable generalizable TCR:pMHC specificity prediction from sequence data [19]. In the absence of sequence data from which binding patterns can be learnt, physics‐based structural methods might be the more promising approach [21].

The challenge with structural approaches is two‐fold: firstly, generating accurate TCR:pMHC complex predictions; and secondly, determining from a structure whether the TCR and pMHC interfaces are sufficiently complementary to bind. Substantial progress has been made in TCR:pMHC complex prediction, and emerging research has begun to tackle the second problem of complex evaluation for specificity prediction.

One method developed to infer TCR specificity from structure data is STAG [20], which transforms the TCR:pMHC interface into a graph representation. The properties of residues are featurised and used as nodes, and the Euclidean distances between residues are encoded as the edge vectors. These graphs are then passed through a bespoke convolutional graph neural network (GNN) [151], which uses an attention‐based convolution operation [20]. The architecture was trained on public specificity data [14], and the TCR:pMHC complex predictions used as the model input were made with TCRpMHCmodels [106]. The benchmark against sequence‐based specificity predictors suggests that STAG performs as well as the best sequence model trained on the same data, NetTCR 2.2 [140].

An alternative approach to training classification models is cognizant that TCR:pMHC complex predictors will always propose a structure regardless of whether the TCR binds the pMHC. Therefore, recent studies have used the structure prediction's confidence metric to distinguish non‐binding from binding TCRs by shuffling the TCRs and predicting the TCR:pMHC complexes [21, 22]. The underlying intuition is that if the model predicts amino acids at the TCR:pMHC interface with low confidence, then these are likely to be difficult to accommodate in the complex, and therefore, are less likely to bind. These approaches are aligned with a broader trend in machine learning research towards verification functions and quality control of generative models and their predictions, usually in the context of synthetic data generation [152, 153].

**FIGURE 3:**
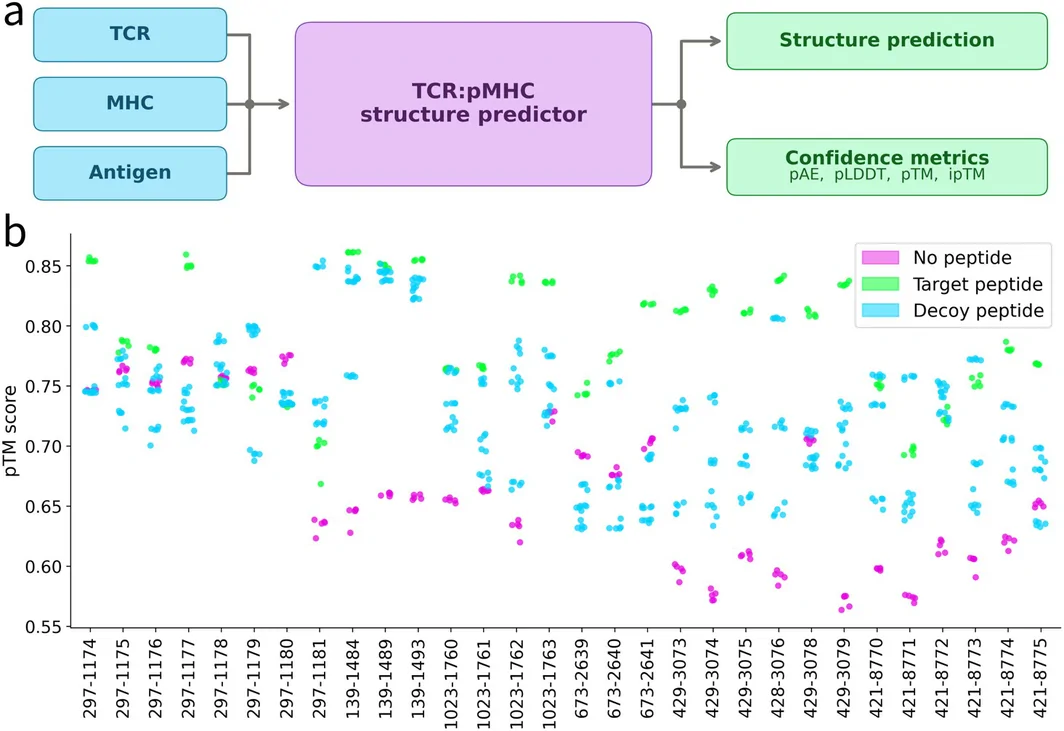
(a) Information flow used to generate TCR:pMHC complex predictions with confidence metrics. Several IMMREP25 methods inferred specificity by ranking or adapting the confidence metrics [37]. (b) pTM (predicted template modeling) uncertainty scores for Protenix‐v1 [28] structure predictions of true and shuffled TCR:pMHC pairs. The ground truth target antigen (green) scores higher than the off‐targets: No antigen, that is, MHC only (magenta) and false antigen, that is, shuffled decoy peptide (blue) in 21/30 cases. Preliminary results generated using the IMMREP25 dataset, *x*‐axis labels indicate TCR identifier and row number in the data.

The first of two recent works to take this approach developed an AlphaFold‐derived TCR:pMHC complex prediction model, TCRdock [21], summarized above in Section 4.2.5. Having developed this model, which produces the structure predictions alongside the standard AlphaFold2 confidence metrics (Figure 3a), the sum of the predicted aligned error (pAE) over all TCR:pMHC residue pairs was calculated. This metric was used to distinguish binding from non‐binding TCRs by predicting TCR:pMHC complexes with peptide decoys as well as the target peptide [21]. The results demonstrate that the confidence estimate can be used to recall target peptides from among the decoys, but that overall structure prediction accuracy must be high in order for the pAE signal to correlate with specificity. Furthermore, the template‐based approach taken by the model limits the predictions to well‐characterized HLA targets. Nonetheless, Bradley et al. have demonstrated the utility of using structure predictions and their confidence metrics for TCR specificity prediction [21].

NetTCR‐struc extends this by training a new evaluation function and combining it with the confidence metric produced by the structure predictor [22]. Similarly to STAG, a GNN is used as the architecture with which structure predictions are processed; however, instead of seeking to directly infer TCR specificity, Net‐TCR‐struc first trains two ensembles of GNNs on predicted structures to estimate the true DockQ scores: one is trained on all structure predictions and one is trained only on those structure predictions with a DockQ score greater than 0.5. The final confidence score, dubbed GNN_AF, is defined as the harmonic mean of the DockQ predictions made by the two GNN ensembles, the global AlphaFold‐Multimer confidence score of the complex, and the arithmetic mean of the pLDDT scores of residues belonging to the CDR loops and peptide. The authors show that their new confidence metric correlates better with DockQ scores than the AlphaFold‐Multimer confidence metric and can therefore better distinguish accurate complex predictions from poor ones. They further demonstrate that their confidence metric can be used to rank the complex predictions and that, in cases where the overall quality of the structure predictions is high, their confidence metric improved the recall of the true TCR:pMHC pair compared to the AlphaFold‐Multimer metric. These results reiterate that the quality of the complex structure prediction is key to inferring specificity from structure.

Reflective of the increasing momentum of structure‐based specificity prediction, the latest IMMREP iteration, IMMREP25, marked the first time that the confidence metrics of TCR:pMHC structure predictions were used by multiple teams as indicators for specificity [39]. Multiple entries achieved a statistically significant improvement in AUC0.1 for previously unseen peptides, where AUC0.1 is defined as the partial area‐under‐the‐curve calculated for false‐positive rates up to 0.1. Of the 15 submissions that yielded improved specificity predictions, 14 had incorporated structure information [39]. The best performing method adapted the TCRdock specificity prediction pipeline to use AlphaFold3 instead of AlphaFold2 [21, 39]. While the improvements on the test set remain modest, with a top AUC0.1 score of 0.601 compared to the random baseline of 0.5, this milestone marks the first time that “de‐novo” specificity prediction methods have generalized beyond the training data.

While the field anticipates the imminent publication of the prediction method protocols, a brief experiment we conducted corroborates the finding that the latest generation of structure predictors produce uncertainty metrics which contain signal for uncertainty quantification. We used Protenix‐v1 [30], an open‐source reimplementation of AlphaFold3 [27], to predict the structures of TCR:pMHC target peptides and shuffled decoy peptides from the IMMREP25 dataset and found that in 21/30 of tested cases, the highest pTM confidence metric corresponded to the target peptide (Figure 3b).

The driver of these promising early results appears to be recent improvements in the accuracy of general protein structure prediction models for TCR:pMHC complexes. Poor accuracy of the complex predictions was highlighted as the key limiting factor by both TCRdock and NetTCR‐struc [21, 140], it would be consistent with these findings if improvements in prediction accuracy translated to improvements in structure‐based specificity prediction. Continued progress in TCR:pMHC structure prediction accuracy may yet unlock a new frontier for TCR specificity prediction.

### 4.4 Outstanding Challenges in TCR and TCR:pMHC Structure Prediction

Despite the development of TCR‐specific prediction models, it remains an open question whether these models outperform general protein models. Recent independent benchmarking suggests that, while improvements have been made, recovery of interactions in the TCR:pMHC interface remains challenging, but that general protein models like AlphaFold2 [26] and AlphaFold3 [27] outperform TCR‐specific models on average [98]. As discussed in Section 2.1, this is perhaps indicative of the paucity of TCR:pMHC structure data available for fine‐tuning and training, which leads to fast overfitting, thereby worsening the generalizability of TCR‐specific models to novel TCR:pMHC data. By contrast, general protein structure prediction models are usually trained on datasets which include TCRs, and it is possible that the additional observed training data on general proteins enables these models to learn information about protein interactions which transfer well to TCRs.

Benchmarking studies indicate that deep learning methods achieve state of the art accuracy on TCR and TCR:pMHC structure prediction, with median backbone errors below 2 Å RMSD across most regions [98]. However, the CDR3α and CDR3β loops remain the most challenging regions of the TCR to predict, reflecting both their intrinsic structural diversity and the limited availability of training data.

While the VDJ‐recombined CDR3β exhibits greater junctional diversity than the VJ‐recombined CDR3α, our recent work demonstrated that CDR3α is at least as difficult to accurately predict, and that, unlike the CDR3L loop, we do not observe the emergence of consistent canonical clusters, which would make the structure of the VJ‐recombined loop easier to predict [25]. Improvements in training set size alone did not yield gains in the average prediction accuracy, though this may also be reflective of the low numbers of TCR structures available for training even when the dataset size is increased [25]. Furthermore, while performance was similar on average, TCRBuilder2+, the model trained on the expanded dataset, showed CDR3 predictive performance improvements for genes for which the number of examples had increased in the expanded training set [25], aligning with related research exploring the generalizability of antibody structure prediction methods [154].

Specifically, analysis of antibody structure predictions made with ABodyBuilder2 [34] revealed that predicted loop structures fall into dense regions of conformational space defined by canonical forms observed in the training data [154]. A study in which the prediction model was retrained whilst withholding canonical conformations revealed the limited ability to extrapolate to novel conformations; however, the inclusion of only a small number of examples in training was sufficient to recover predictive capacity. Transferring these insights from antibodies to TCRs suggests that deep learning structure predictors are likely to continue to struggle to predict completely unseen conformations of the TCR CDR loops, but that directed experimental determination of TCR structures with as yet unseen VJ‐gene pairs could yield improvements in structure prediction accuracy.

While this is a tall ask for antibodies, where the combinatorial diversity of the heavy chain recombination is very large, for TCRs this would amount to approximately 2000 structures, provided novel TRAV‐TRAJ and TRBV‐TRBJ chain pairings are made every time. By further selecting on the basis of sequence similarity to maximize the observed diversity, this number could potentially be reduced further and could actively counteract the dataset biases observed in TCR structure data. If the TCR structure determination experiments were conducted at high throughput and low cost using a standardized and automated protocol, it could be possible to structurally characterize the complete VJ‐gene pairing space in future.

A caveat is that CDR3α and CDR3β loop conformations may not be independent of each other, with evidence of inter‐loop interactions emerging in some cases, as well as a shift in relative orientation of the Vα and Vβ domains [155]. Expanding the search space to include all TRAV‐TRAJ/TRBV‐TRBJ combinations yields > 2 × 10^6^ TCRs that would need to be crystallized, which is approximately 10× more than all the data deposited in the PDB as of January 2026 and so is not currently practically feasible. Solving the VJ‐gene space for each chain independently is a more pragmatic approach which could yield TCR structure prediction improvements.

Furthermore, as discussed in Section 3.3, the CDRs of TCRs are often flexible and can exhibit a degree of induced fit upon binding to the pMHC [80, 156, 157, 158, 159]. The induced fit of TCR loops observed upon binding implies that structure predictors would benefit from information about the antigen to predict complementary TCR structures, and that a hypothetical, perfect TCR structure predictor would require information about the antigen to predict a single conformation. In addition, current structure prediction models are predominantly trained on TCRs in complex with antigen, since these are considerably more abundant in the existing data [24]. If the antigen significantly influences the eventual bound structure of the TCR, then it is likely that current model predictions will struggle to accurately predict *apo* TCR structures, a challenge we recently discussed for antibodies [160].

In prior work, we conducted an MD‐case study to investigate the physical plausibility of different predicted CDR3 loop conformations [25]. However, the simulations were inconclusive as regards whether a specific conformation was “correct” or whether multiple loop conformations are valid for *apo* TCRs [25]. MD datasets such as FTCRDab (Section 3.3) collect information about TCR loop flexibility and have been used to train flexibility prediction tools, as well as structure predictors which explicitly seek to predict multiple conformations, thereby capturing loop flexibility [82]. Training models on this data may yet improve the predictions of both structure and flexibility, and there is precedent for improving the sampling of physically plausible structure predictions by training on MD simulations [161, 162, 163].

Alternatively, flexibility of protein structure predictions has been derived from model confidence metrics, or explored by sampling multiple predictions [164, 165, 166]. While these methods are able to capture notions of underlying true flexibility, it remains difficult to disentangle errors in the prediction from truly flexible regions, and confidence metrics, such as pLDDT, have been used to infer both [26, 165]. Especially in the context of using confidence metrics to extrapolate biologically relevant characteristics, such as TCR specificity, there is likely merit in better disentangling when confidence metrics are indicative of predictive errors and when they are indicative of true flexibility. The statistical field of uncertainty quantification may provide some useful methods which transfer to this scenario [167].

## 5 Forward Perspectives

### 5.1 Repertoire‐Scale TCR Structure Prediction

#### 5.1.1 Curating Paired‐Chain TCR Repertoire Data Enables Repertoire‐Scale Structure Prediction

Structure predictions can be used to add a new dimension of information to deep, highly diverse TCR‐repertoire data, and can reveal patterns that cannot be determined from sequence and enrichment data alone [25, 38]. Given that TCRs are heterodimers, paired‐chain sequence information is requisite for structural modeling of TCR Fvs. In recent work, we predicted the structures of over 1.5 million TCR sequences deposited in OTS, a paired‐chain TCR repertoire database we curated and published [168].

Specifically, OTS was built to address the absence of a dedicated paired‐chain TCR sequence repository by aggregating and consistently processing 10× single‐cell sequencing data from 50 studies and more than 75 individuals into 5.35 million redundant (1.63 million non‐redundant) predominantly human, full‐length paired TCR sequences [168]. The uniform curation pipeline, which we extended from the analogous paired OAS antibody database, annotates each sequence with metadata and can be explored via a web application. We also used OTS as the training corpus for the paired‐chain TCR language model, TCRLang, which provides embedding representations and a TCR design method.

In tandem with the development of extremely high‐throughput TCR structure predictors, capable of predicting a structure in under 1 s on a modest GPU, the release of OTS enabled the first repertoire‐scale prediction of TCR structures [25]. The resulting dataset of over 1.5 million TCR structures, predicted using TCRBuilder2+ [25], constitutes the largest publicly available repository of TCR structure information. Our analysis of this dataset helped reveal the equivalent structural diversity of the CDR3α relative to the CDR3β (Section 4.1.3), and we expect the size of the dataset to help unlock the application of deep learning methods to TCR structures.

While prediction accuracy remains the primary benchmark against which TCR structure prediction tools are evaluated, operating at repertoire scale introduces speed as an equally fundamental constraint. In therapeutic and engineering contexts where the number of TCR candidates being developed typically ranges from tens to hundreds, runtimes of minutes to hours per structure are routinely acceptable, and this is the regime for which most current predictors have been optimized. By contrast, repertoire‐scale analyses involve much larger numbers of sequences: a single deep‐sequenced paired‐chain repertoire from one individual may contain millions of unique clonotypes [169]. At a throughput of one prediction per second using TCRBuilder2+, predicting the structures of a single repertoire containing 1 million sequences would take approximately 12 days without structure refinement or any downstream analysis of the structures [25, 34]. This renders the incorporation of structure prediction into routine repertoire analyses at best impractical, and at worst infeasible, depending on the compute hardware. Hence, advances both in prediction accuracy and speed are crucial to improve and enable repertoire‐scale structure analysis.

Furthermore, paired‐chain repertoire sequencing is enabled by single‐cell methods, which preserve α‐β pairing and therefore provide complete TCR sequences [168, 170]. This makes paired‐chain data especially valuable for “mechanistic” studies: they permit antigen‐specific receptors to be analyzed in terms of their full interface, and they make it possible to integrate repertoire analysis with structural and specificity modeling. However, compared to bulk‐sequencing methods, single‐cell protocols are substantially more expensive and sequence at lower throughput [170, 171]. This is of particular concern for associative studies which rely on the observation frequencies of sequences: reduced sequencing depth leads to lower sensitivity to moderate clone expansion and worsens the signal‐to‐noise ratio of sequencing [171]. The throughput and cost of single‐cell sequencing is likely to continue improving, and concurrently developed statistical methods for chain pairing from bulk‐sequence data across multiple wells provide a compelling, low‐cost alternative for deep, paired‐chain sequencing [171, 172]. Nonetheless, at present, bulk‐sequencing protocols yield the deepest repertoire data, and are well‐suited to clonotype discovery studies for which chain pairing is not critical [173, 174].

#### 5.1.2 Bulk Sequencing Repertoire Data Is Not Currently Used for TCR Structure Prediction

Bulk TCR sequencing enables deep repertoire profiling by sequencing collections of cells from biopsies or blood samples, yielding 10^4^ to 10^6^ TCR clonotypes per sample, whereby a clonotype is typically defined as a unique sequence of a β chain, or, less commonly, an α chain [174, 175]. Analysis of the clonotype frequency yields insights into the immune state of the sampled tissue [174, 175]. Bulk repertoire analysis is therefore well suited to answering questions of association: it can identify expanded clones, recurrent motifs, and public clonotypes shared across individuals or cohorts, which can be informative about infection, vaccination, malignancy, or autoimmunity [174].

However, bulk repertoire sequencing does not provide chain pairing information [171]. Given that α and β chains pair combinatorially and that a β chain may be compatible with multiple α chains, a β‐chain sequence on its own does not reliably define a unique receptor, but rather a set of possible receptors with distinct paratopes [176]. Bulk sequence data are therefore well suited to identifying candidate receptors from enriched clones, but are not conducive to reconstructing the complete binding interface required to investigate the molecular mechanism of pMHC recognition which is distributed across both chains [15, 57].

Furthermore, existing TCR structure prediction tools use paired‐sequence inputs [21, 25, 36, 37]. Given that the majority of repertoire sequencing data is single‐chain, typically beta‐chain only, most sequence data collected at repertoire scale cannot currently be used for structural modeling of TCRs or TCR:pMHC interfaces [168, 177].

For antibodies, we addressed this limitation by creating HeavyBuilder, a version of ABodyBuilder2 which predicts the heavy chain structure in the absence of the light chain [178]. This enables high‐throughput modeling of heavy‐chain‐only antibody repertories, which are substantially more abundant than paired‐chain antibody data: as of April 2026, OAS, our database of antibody repertoire sequences, contained over 3 million paired‐chain antibody sequences, and over 2 billion unpaired antibody sequences, corresponding to approximately one paired chain data point for every 665 unpaired sequences [179]. Having trained HeavyBuilder, our subsequent analysis of repertoire data of Covid‐19 cohorts revealed structural convergence beyond clonal lineages, demonstrating that even partial information about the structure of the antibody CDRs provides orthogonal insight to sequence and expansion data alone [178].

A single‐chain structure prediction tool has not yet been developed for TCRs, but could reveal similar structural convergence patterns. However, the utility of a single chain model may prove to be lower than for antibodies; for antibodies evidence suggests the heavy chain drives antigen recognition over the light chain [83, 101], whereas the TCR:pMHC interface is more sensitive to small sequence changes, and pMHC specificity is often determined by both the CDR3α and CDR3β [180].

In summary, while progress can still be made towards generating high‐throughput paired chain TCR repertoire data, both the quantity and curation of this data has enabled the prediction of TCR structures at repertoire‐scale [25]. As the prediction accuracy and speeds improve, a new dimension of structural analysis will be unlocked for repertoires.

### 5.2 Opportunities Arising From Antibody Structure Analysis

While substantial research has been conducted to characterize TCR and TCR:pMHC complex structures leading to insights into TCRs and their binding and signaling mechanisms [15, 67], the field has not yet observed the widespread adoption of tools which use structural information to infer TCR properties at scale. Computational tools developed for antibodies provide precedent and have demonstrated the types of novel insight that can be gained; antibody‐specific tools could therefore act as a source of inspiration for TCR‐software development.

Structure data is particularly useful for the characterization of physicochemical properties, which has enabled profiling and filtering of antibodies on the basis of desirable properties [181]. Our Therapeutic Antibody Profiler (TAP) used the predicted structures of post‐Phase I clinical‐stage antibodies to characterize the distributions of developability‐associated features and, having established these, evaluated and filtered candidate antibodies by predicting their structure and calculating their features [181]. Building on this work, TAP2 recalculated the developability thresholds using updated datasets and ImmuneBuilder, the high‐throughput deep learning antibody structure prediction tool [34, 182]. Similar structure‐derived metrics for common TCRs could be revealing for the development of TCR‐based soluble therapeutics and may yield corresponding novel insights for developable TCR properties.

Predicted TCR structures enable the training of machine learning models that operate directly on three‐dimensional features, rather than sequence alone, and therefore, as discussed previously, have the potential to capture more generalizable principles of antigen recognition [20]. In this setting, structure prediction acts as an enabling layer, allowing TCR sequence datasets to be projected into a representation that is more closely aligned with mechanism. For instance, although the relationship between structure and specificity remains complex and incompletely understood, examining the distribution of structural features across TCR repertoires may reveal constraints and patterns that are not apparent from sequence alone.

A suite of tools developed for antibodies, SPACE and the ensuing SPACE2, cluster antibodies by CDR loop structure, rather than by sequence [38, 183]. These algorithms calculate structure‐based distance metrics across thousands of antibody structures and proceed to cluster the antibodies by similarity. Analysis enabled by SPACE and SPACE2 revealed that structure is often conserved across epitope‐specific antibodies, even when these originate from different clonotype lineages and are sequence diverse [38, 183]. This structural convergence was exemplified using the Coronavirus Antibody Database (CoV‐AbDab) [184] and suggests that immune receptor convergence transcends clones, thereby confirming that structure data contains sequence‐orthogonal information about epitope specificity. No algorithms have yet been developed which map epitope specificity or disease states to clusters of structurally similar TCRs, though this has been explored for sequences [124, 125]. The development of high‐throughput structure predictors and annotated databases such as OTS have made this type of analysis possible, and could reveal as yet undiscovered clusters of structurally convergent TCRs [25, 168].

Other examples of learning from antibody structure predictions are antibody design tools, including AntiFold [185] and AntiDIF [186]. We expand upon deep‐learning design methods in Section 5.3.2; briefly, AntiFold fine‐tunes ESM‐IF [187], an inverse‐folding model, and demonstrates high sequence‐recovery rates, particularly in regions likely to be key for epitope specificity. AntiDIF builds on this work but improves the diversity of designed sequences by fine‐tuning a diffusion model. Excitingly, deep learning structure‐based design pipelines for TCRs and pMHC binders are emerging and improving as the field of “de‐novo” protein design progresses.

### 5.3 The New Frontier of In Silico TCR Design

#### 5.3.1 Structure‐Based TCR Engineering

Rational design of TCRs is moving from being an experimentally guided discipline to a computationally guided one. Pioneering work used computational mutagenesis and energetics software, such as was integrated in the ZAFFI protocol [188], applied to crystal structures of TCR:pMHC complexes, to identify candidate point mutations in amino acids to improve the binding affinity of TCRs to antigen [188, 189, 190]. Surface plasmon resonance experiments were used to confirm the productive mutations and have shown that combining affinity‐improving mutations can lead to 100‐ to 400‐fold improvement in affinity to the antigen [188, 189]. Alternative approaches have approximated the change in dissociation constant using steered MD simulations, combined with free‐energy estimations [191].

A pre‐requisite for these structure‐based engineering approaches is an accurate starting structure such that the local energetics surrounding point mutations can be evaluated. Mutating several residues from the known structure can cause structural deviations, worsening the free energy estimates and, by extension, the reliability of the proposed mutations. However, the maturation of sequence to structure predictors (see Section 4.4) has made structure readily available, and also enabled broadening of the scope of design from amino‐acid mutations to full sequences.

#### 5.3.2 “De‐Novo” TCR Design

In parallel to developments in structure prediction, machine learning modeling of language has accelerated up to the point where artificial intelligence (AI) tools have become widespread and are used for many tasks that were previously done by humans. AI tools and chatbots are built upon Transformer‐based large language models and similar approaches have been applied to protein sequences [114]. These models are known as protein language models (PLM), and they can encode relevant information about protein sequences, including structure information, and be used to generate novel protein sequences.

General protein models such as the ESM models [114, 192] are trained on all protein sequences, while TCR‐specific models have been trained exclusively on TCR sequence data. TCRlang is a TCR language model trained on paired sequence data curated in OTS (Section 5.1.1) [168], while TCRdesign is a model which generates just the TCR CDR3β loop conditioned on an antigen such that the TCR is likely to bind [193]. Using protein language models for sequence generation is an approach to TCR design which avoids having to predict structures. Specifically, in the model developed by Li et al. [193], the antigen sequence is embedded and used as the key and value in a cross‐attention module of a Transformer‐like architecture and used to generate the CDR3 sequence of the β chain. While this avoids the errors which compound from structure prediction in structure‐based design pipelines, it is not yet clear that these sequence‐based design methods are able to avoid the dataset biases and generalization difficulties observed in sequence‐based specificity predictors.

Simultaneous to language model developments, access to accurate computational structure predictors has enabled training of so‐called “inverse folding models” [187, 194]. These models are trained on protein structure backbones to predict distributions of sequences that refold into the desired structures, and models such as ProteinMPNN [194] and ESM‐IF [187] have been widely adopted. Both models produce a distribution for the amino‐acid identities in the sequence after embedding the backbone structure using a GNN. Similar to language models, final candidate sequences can then be sampled auto‐regressively from the distribution. These predicted sequences are then evaluated using a structure prediction tool such as AlphaFold to refold the structure and compare it to the original input backbone.

Inverse folding models require prior knowledge of the protein structure that the sequence should fold into, to address this, backbone generation models such as RFDiffusion [195, 196] have been developed, which define residue positions without inferring their amino‐acid identity. These generated backbones can then be used as input to inverse folding models to retrieve sequences likely to fold into the designed structure. As such, “de novo” protein design is a three‐stage process: first, the desired backbone structure is generated, then this structure is inverse‐folded to yield candidate sequences, and finally the proposed sequences are re‐folded such that the resulting structure can be compared to the desired backbone and the prediction confidence used as a quality‐control metric.

Inverse‐folding for TCRs using ProteinMPNN and ESM‐IF has been shown to recover native TCR sequences at the pMHC interface, and that these methods improved pMHC‐dependent recovery compared to RosettaDesign, an energy‐based design method [197]. Here, the authors used TCR:pMHC crystal structures withheld from the ESM‐IF and ProteinMPNN training set and evaluated the recovery rates of the true CDR loop sequences; however, the exact TCR:pMHC backbone structure is a pre‐requisite in this framework to enable the design of the inverse‐folded sequence. More recently, Motmaen et al. addressed this by using a fine‐tuned version of AlphaFold2 to predict diverse conformational poses of TCR:pMHC complexes, and then using ProteinMPNN to produce candidate sequences for the CDR loops [198]. This design pipeline replaces RFDiffusion with AlphaFold2 for backbone design and therefore requires initial TCR sequences with which the structure predictions are seeded. This architecture choice was motivated by the observation that a fine‐tuned version of RFDiffusion produced higher proportions of reverse binding TCR:pMHC backbone structures and higher error rates during re‐folding. After iterating through the design pipeline and applying a bespoke confidence metric as a quality control for the designs, experimental validation showed that the designed TCRs were specific binders for six of the nine pMHC targets. While many of the binders were sufficiently specific to the target peptide when compared to off‐target peptides, some of the designs induced T‐cell signaling upon binding to MHC with no peptide. This suggests that the designs have not yet fully captured the sensitive specificity of natural TCRs to both the peptide and the MHC and increases the risk of off‐target reactivity. This work constitutes a step towards in‐silico design of the entire TCR paratope and the experimental results are supportive of the efficacy of computational TCR design for binder design, while revealing that specificity remains an area for improvement.

**FIGURE 4:**
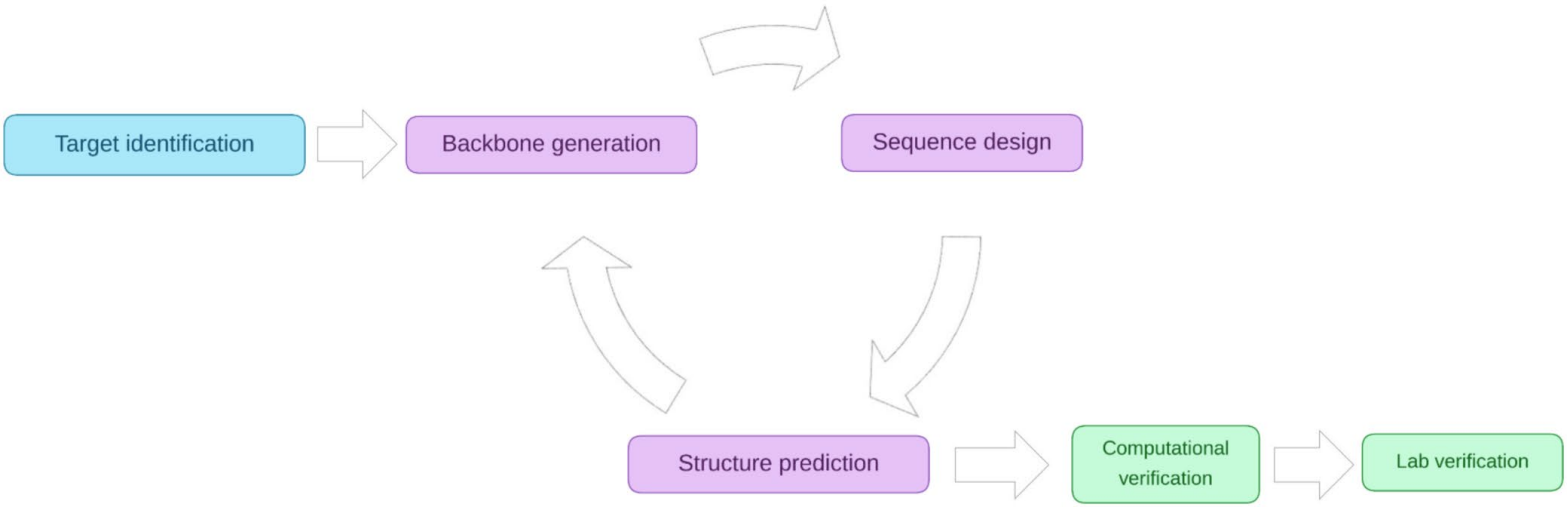
Flow diagram of a “de novo” protein design pipeline used to design TCRm minibinders to pMHC [56, 57, 58]. After target identification, a backbone structure is generated, conditioned on the target. This backbone is “inverse folded” to design an amino‐acid sequence, and the structure prediction of that sequence is validated against the desired backbone. Several rounds of design are augmented with computational and experimental verification of binding and other desirable properties.

Recently, this design pipeline (Figure 4) has been adopted to design novel TCR‐mimic (TCRm) binders to pMHC targets, with specificity assays confirming high‐affinity, target‐specific binding [58, 59, 60]. The low off‐target cross‐reactivity profiles of these designs are promising; however, current backbone designs constitute helical bundles, substantially differentiating these protein binders of pMHC from designed TCRs. HLA‐specific minibinders have been incorporated into CAR constructs and shown to confer peptide‐specific T cell activation and tumor cell killing [59, 60], extending demonstrations with non‐pMHC targets such as CD20 in the “Bits to Binders” competition, where submitted designs achieved hit rates of 0.6%–38.4% [199]. Relative to engineered TCR therapies, these TCRm‐CAR approaches benefit from the elimination of endogenous TCR chain mis‐pairing risk and co‐receptor independence, enabling both CD4+ and CD8+ T cells to contribute cytotoxicity [200, 201]. Nonetheless, as with other CAR‐T therapies, they may be constrained by the reduced signaling sensitivity of CAR constructs compared to the native TCR/CD3 complex, which could require higher pMHC surface densities for T‐cell activation [202, 203].

#### 5.3.3 Alternatives and Latest Developments in Protein Design

Protein design is an active field of research at the intersection of deep learning and computational structural biology. As such, some models developed for general protein design have yet to be applied to TCRs. Currently, two alternative methods exist which differ from the backbone generation and inverse folding pipelines described above.

“Hallucination” provides an alternative framework for structure‐based design and encompasses methods that predict structures from random initial sequences and iteratively optimize the sequences by repeated sampling [204]. BindCraft, the current state‐of‐the‐art for hallucination‐based protein design, iteratively optimizes the sequence via gradient descent, back‐propagating from a “design loss” term calculated from AlphaFold2 structure predictions of the sequence [205]. Instead of calculating the loss gradient with respect to the network parameters as is done to train the network, BindCraft calculates the loss gradient with respect to the input sequence. This gradient is then applied to the input sequence iteratively to reduce the design loss until a final set of optimized sequences has been designed. BindCraft also uses additional sampling and optimization strategies for properties such as solubility and to ensure sufficient exploration of sequences. The authors show that 10%–100% of the designed proteins bind the target, a competitively high hit rate for a computational design model, but note that back‐propagating through AlphaFold2 is computationally expensive. To date, BindCraft has been used to design general protein binders, but this method has not yet been extended to TCRs.

Finally, recent work has demonstrated the utility of sequence‐structure co‐design methods [206, 207, 208, 209, 210]. These models seek to merge the two design steps: structure generation and sequence infilling, into a single prediction. Broadly, approaches either use a generative model to produce the structure and sequence simultaneously [206], or use an all‐atom representation to generate structures with atom positions which encode residue identities [207, 208]. These methods have achieved experimentally validated binder hits by conditioning the generative process on known targets and are currently considered the state‐of‐the‐art for computational design [210, 211]. While sequence and structure co‐design methods have been developed for antibodies [212, 213, 214, 215], these methods have not yet been applied to TCRs.

## 6 Concluding Remarks

The maturation of computational structure prediction has enabled fast and accurate prediction of TCR and TCR:pMHC complex structures. While the CDR3 loops of both the α and β chain remain the most difficult to model, improvements in general protein structure prediction will likely continue to translate to TCR:pMHC complex prediction, particularly as the focus of researchers shifts towards accurate prediction of protein:protein interfaces. For TCRs, given both the flexibility of the CDR loops and the utility of prediction confidence metrics for specificity prediction, a better understanding of structure prediction confidence metrics could prove insightful [39]. In particular, disentangling flexibility from prediction errors could enable more reliable use of confidence metrics for specificity determination, given that prediction error is currently the reported bottleneck [21, 22].

At present, TCR‐specific structure predictors perform similarly to general protein structure predictors, likely because general models also train on TCR data, and TCR data is relatively sparse [98]. Observations that deep learning models need few examples of a loop conformation in order to make predictions of a novel conformation [154], alongside bias in TCR structure data [25] suggest that generating the structures of TCRs with as yet unsampled TRAV‐TRAJ and TRBV‐TRBJ gene pairs could yield improvements in structure prediction. This could represent approximately 2000 structures and could be crystallized and resolved by a consortium of researchers, as is currently being undertaken for protein‐ligand structures [216].

Structure predictions of TCR:pMHC complexes may be the key to unlocking TCR specificity prediction, for which the current consensus is that too little sequence data exists to train generalizable models [17, 19]. Analysis of the failure modes of structure‐based specificity prediction revealed the importance of accurate TCR:pMHC complex predictions [21, 22]. This implies that any improvement in TCR and TCR:pMHC structure prediction accuracy will yield improvements in TCR specificity predictions “for free,” provided the reliability of the uncertainty metrics is retained, and provides a compelling argument for seeking to improve complex predictions further.

Another domain in which improvements in TCR structure prediction will unlock improvements is TCR design. Both TCR engineering with residue substitutions and “de novo” design using hallucination or inverse‐folding methods are reliant on structure prediction to validate their design, generate training data, and, in the case of hallucination, to generate predictions in the first place. Recent work demonstrated in vitro binding of designed sequences with the use of “mini‐binders”, non‐TCR engagers of pMHC [60], as well as with TCRs [198]. While some concerns around cross‐reactivity remain, it is clear that TCR structure prediction has already enabled, and will continue to enable, substantial progress in TCR‐derived therapeutics design.

Furthermore, access to accurate TCR structure predictors increases the value particularly of paired TCR sequence data, as more information can now be gleaned from it. The curation of over 2.3 M paired‐chain TCR sequences in our OTS database provides the first repertoire‐scale dataset that characterizes the complete paratope of TCRs [168]. Coupled with advances in TCR structure prediction, this dataset unlocks a new type of structural repertoire analysis, which has been shown to yield orthogonal insights for antibodies [38, 178, 181, 182, 183]. Speed remains a key issue for repertoire‐scale structure prediction: even if one structure can be predicted per second, a repertoire sample containing 10^6^ sequences would take approximately 12 days.

In conclusion, the impact that deep learning has had and continues to have on structural immunoinformatics is tremendous: since the publication of AlphaFold2 [26] methods have developed at an incredible pace, providing an exhilarating environment in which to conduct computational TCR research. Multiple exciting avenues of research are emerging, ranging from structural characterization of repertoire data to “de‐novo” specificity prediction, to pMHC binder design, and we anticipate substantial progress in the coming years.

## Funding

This work was supported by the Engineering and Physical Sciences Research Council, EPSRC, grant EP/S024093/1 and Immunocore Ltd via SABS R3 CDT.

## Conflicts of Interest

C. M. D. discloses membership of the Scientific Advisory Board of Fusion Antibodies and AI Proteins, as well as being a founder of Dalton. All other authors declare no conflicts of interest.

## Data Availability

Data sharing not applicable to this article as no datasets were generated or analyzed during the current study.
